# New Antibiotics Against Multidrug-Resistant Gram-Negative Bacteria in Liver Transplantation: Clinical Perspectives, Toxicity, and PK/PD Properties

**DOI:** 10.3389/ti.2024.11692

**Published:** 2024-02-01

**Authors:** Andrea Lombardi, Laura Alagna, Emanuele Palomba, Giulia Viero, Anna Tonizzo, Davide Mangioni, Alessandra Bandera

**Affiliations:** ^1^ Department of Pathophysiology and Transplantation, University of Milano, Milan, Italy; ^2^ Infectious Diseases Unit, IRCCS Ca’ Granda Ospedale Maggiore Policlinico Foundation, Milan, Italy

**Keywords:** liver transplantation, BL/BLI, multidrug-resistant microorganisms, antimicrobial stewardship, metallo-beta lactamases

## Abstract

Antimicrobial resistance is a growing global health problem, and it is especially relevant among liver transplant recipients where infections, particularly when caused by microorganisms with a difficult-to-treat profile, are a significant cause of morbidity and mortality. We provide here a complete dissection of the antibiotics active against multidrug-resistant Gram-negative bacteria approved over the last years, focusing on their activity spectrum, toxicity profile and PK/PD properties, including therapeutic drug monitoring, in the setting of liver transplantation. Specifically, the following drugs are presented: ceftolozane/tazobactam, ceftazidime/avibactam, meropenem/vaborbactam, imipenem/relebactam, cefiderocol, and eravacycline. Overall, studies on the safety and optimal employment of these drugs in liver transplant recipients are limited and especially needed. Nevertheless, these pharmaceuticals have undeniably enhanced therapeutic options for infected liver transplant recipients.

## Introduction

A significant challenge facing humankind in the 21st century is antibiotic resistance, and liver transplantation (LTx) is not immune to this threat [1]. Indeed, it is well-known how infections frequently occur in liver transplant recipients (LTR), with about 55% of them experiencing an infection within 12 months after transplantation [2]. This translates into relevant mortality, with infections being the most frequent cause of death 30–180 days after LTx [3]. Unfortunately, an increasing amount of these infections are caused by multidrug-resistant (MDR) bacteria [4]. Among them, MDR Gram-negative bacteria (MDRGNB) are responsible for most infections [5–8].

Colonisation by MDRGNB is a common condition in LTR, which reflects the long clinical history and exposure to antimicrobials and healthcare settings of these patients. The gastrointestinal tract represents the reservoir of MDRGNB, where resistance mechanisms are selected, maintained, and exchanged between species, leading to the so-called “gut resistome” [9].

Colonisation rates among LTR mirror the increasing frequencies observed worldwide in the general population [10]. This is reflected in an increased incidence of infections due to MDRGNB, with infection rate due to ESBL-producing Enterobacterales (ESBL-E) among colonised patients seven times higher than in non-colonised [11]. Similarly, carbapenem-resistant Enterobacterales (CRE) infection rates have been estimated at 18.2% and 2% among colonised and non-colonised LTR, respectively [12].

Regarding outcomes, increased mortality has already been highlighted for liver transplant candidates on the waiting list colonised by MDRGNB compared to non-colonised (HR = 2.57, *p* < 0.0001) [13]. The same relevance has also been confirmed in the post-transplant setting, with patients developing post-transplant CRE infection having a 50% less chance of survival versus those uninfected (0.86, 95% CI, 0.76–0.97 vs. 0.34, 95% CI 0.08–1.0, *p* = 0.0204) [14] and several other studies confirming the role of MDRGNB in hampering survival [15, 16]. The same negative outcome has been associated with infection due to MDRGNB not belonging to the Enterobacterales genus, with recipients having carbapenem-resistant *Acinetobacter baumannii* (CRAB) infection showing a 60-day mortality of 46.4%, significantly higher than the one displayed by those not infected [17].

Notably, in the recent past, when the therapeutic armamentarium was limited to old or side-effects-prone antibiotics, colonisation by CRE was suggested as a reason for withdrawal from transplantation list, thus severely impacting the life expectancy of patients needing LTx [18].

Luckily, since 2014, several new antibiotics have entered the market: ceftolozane/tazobactam (C/T), ceftazidime/avibactam (CZA), meropenem/vaborbactam (MVB), imipenem/cilastatin/relebactam (I-R), cefiderocol (FDC), and eravacycline (ERV). They are an older beta-lactam (BL) plus a new beta-lactamase inhibitor (BLI) (CZA, MVB, I-R), a new BL plus an older BLI (C-T), a new siderophore cephalosporin (FDC), and a new tetracycline (ERV). Recently published guidelines from scientific societies regulate the use of these molecules in the general population [19–21]. We provide a complete dissection of these new molecules, focusing on their activity spectrum, toxicity profile and pharmacokinetic/pharmacodynamic (PK/PD) properties, including therapeutic drug monitoring, in LTx.

**FIGURE 1:**
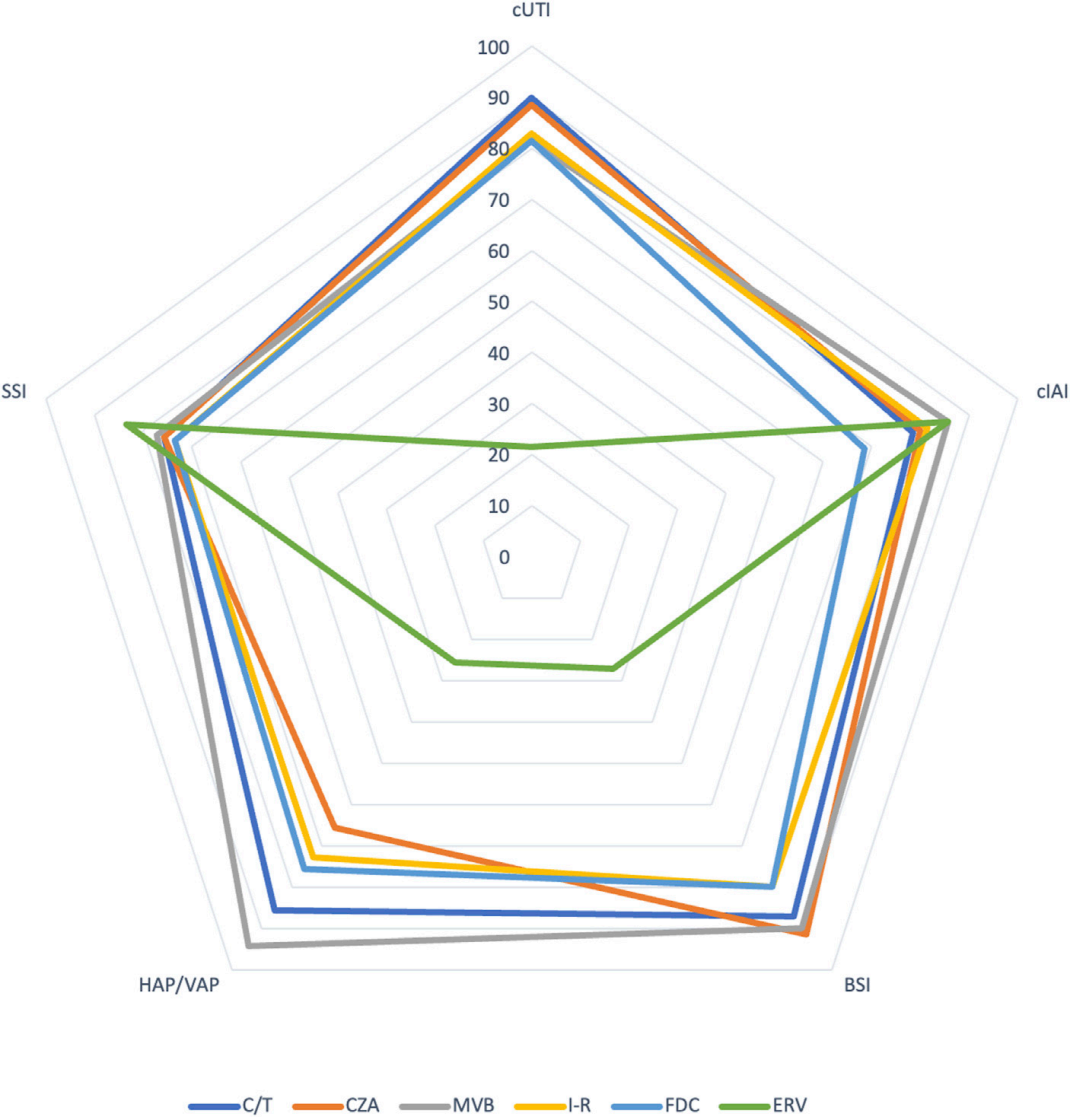
Comparison of propensity to new antibiotic use in common infectious conditions among LTR according to authors’ opinion (Personal view). Based on a hypothetical fully susceptible microorganism toward the antimicrobial considered. (0 = totally against use, 100 = totally in favour of use) (HAP/VAP: hospital-acquired pneumonia/ventilator-associated pneumonia, BSI: bloodstream infection; cIAI: complicated intraabdominal infection; cUTI, complicated urinary tract infection; SSI: surgical site infection; C/T: ceftolozane/tazobactam; CZA: ceftazidime/avibactam; MVB: meropenem/vaborbactam; I-R: imipenem/relebactam; FDC: cefiderocol; ERV: eravacycline).

Table 1 provides an overview of common MDRGNB resistance mechanisms/profiles and the corresponding activity of new antibiotics. Figure 1 compares the propensity of new antibiotic use in common infectious conditions in LTR according to the authors’ opinions (personal view).

**TABLE 1:** Activity spectrum of recently approved antibiotics against multidrug-resistant Gram-negative bacteria.

Antibiotic (year of approval by EMA)	ESBL	KPC	MBL	Amp-C	Oxa-48	P.aer-DTR	CRAb
Ceftolozane/tazobactam (2015)	✓	✗	✗	✓	✗	✓	✗
Ceftazidime/avibactam (2016)	✓	✓	✗	✓	✓	✓/✗	✗
Meropenem/vaborbactam (2018)	✓	✓	✗	✓	✗	✗	✗
Imipenem/relebactam (2020)	✓	✓	✗	✓	✗	✓	✗
Cefiderocol (2020)	✓	✓	✓	✓	✓	✓	✓
Eravacycline (2018)	✓	✓	✓/✗	✓	✓	✗	✓/✗

ESBL: extended-spectrum beta-lactamases; KPC: *Klebsiella pneumoniae* carbapenemase; MBL: metallo-beta-lactamase; Amp-C: AmpC β-lactamases; OXA-48: OXA-48, carbapenemase; P. aer-DTR: difficult-to-treat *P. aeruginosa*; CRAb: carbapenem-resistant *Acinetobacter baumannii*.

## Ceftolozane/Tazobactam (C/T)

### Activity Spectrum

C/T is an association between a fifth-generation cephalosporin, ceftolozane, and a well-known BLI, tazobactam [22]. Ceftolozane displays activity against Gram-negative bacilli, including those that produce β-lactamases. However, it is compromised by ESBLs, whose actions are overcome by adding tazobactam. Unlike other BLI such as avibactam, vaborbactam and relebactam, tazobactam does not inhibit carbapenemases, so C/T should not be used to manage CRE [23]. Instead, ceftolozane has an excellent capacity for penetration through porin canals and evades most resistance mechanisms displayed by *P. aeruginosa*, including efflux pumps, modification of penicillin-binding proteins and Amp-C expression. Due to these properties, C/T is primarily active against *P. aeruginosa* and ESBL-E [24].

C/T has been approved for the treatment of complicated urinary tract infections (cUTI) [25], complicated intra-abdominal infections (cIAI) [26] and ventilator-associated bacterial pneumonia (VABP) [27]. The licenced dose of C/T in patients with normal renal function is 1.5 g every 8 h for cUTI [4] and cIAI [5] and 3 g every 8 h for VABP [6]. Of note, dosages should be reduced in patients with impaired renal function.

### Ceftolozane/Tazobactam in Clinical Trial and its Potential Application in SOT Recipients

Overall, C/T appears to be a novel BL/BLI combination particularly effective against serious infections caused by MDR and XDR *P. aeruginosa*, and most of the current studies address its use in this setting with promising clinical outcomes. However, there is little data on solid organ transplant (SOT) recipients and even less on LTR.

A good outcome for the use of C/T in *P. aeruginosa* infections with limited treatment options is reported in a multicentre retrospective study of 263 patients, achieving a composite clinical success in 70% of patients, confirmed in the SOT subgroup (60.8%, 4/23 patients). Only two patients were LTR in this study, and one in two achieved clinical success [28]. Similarly, Bassetti et al. performed a multicentre nationwide study of C/T for treating severe *P. aeruginosa* infections, with 83% of patients having a successful clinical outcome at the end of treatment. There were 11 SOT recipients in the population, but neither the transplanted organ nor the disaggregated outcome is available [29]. The efficacy of C/T in the treatment of MDR *P. aeruginosa* and MDR Enterobacterales infections is also demonstrated by Ronda et al., who describe 30.1% treatment failure and 30-day and 90-day all-cause mortality of 8.6% and 17.2%, respectively. Interestingly, most of the 96 episodes analysed occurred in immunosuppressed patients (57.9%), of whom 17 (22.4%) were SOT recipients, including one LTR [30].

Promising news for LTR treated with C/T comes from real-world data, as reported by Escolà-Vergé et al. in their review of cIAI caused by MDR *P. aeruginosa*, which presents the cases of a 70-year-old LTR with liver abscesses and a 44-year-old LTR with septic shock due to cholangitis, with both patients reaching clinical cure and microbiological eradication [31].

### Adverse Events and Limitations

There is limited information on using C/T with immunosuppressive agents in SOT recipients. Ceftolozane is not expected to have clinically significant drug-drug interaction as it is neither a substrate nor a modulator of the cytochrome P450 system at therapeutic concentrations. Instead, tazobactam is a substrate of the organic anion transporters 1 and 3, and the coadministration of drugs that may inhibit these transporters may increase its plasma concentrations. In a study evaluating the physical compatibility of C/T with selected intravenous drugs during simulated Y-site administration, Thabit et al. found that C/T was incompatible with cyclosporine due to turbidity changes [32].

C/T is generally well tolerated, with the most common adverse events being nausea, vomiting, and diarrhoea [3]. It is almost eliminated as an unchanged form by the renal route (92%) and is not extensively metabolised by the liver, making it a good candidate for use in LTR [2].

### Key Messages

Despite the paucity of data on the use of C/T in LTR, the available studies suggest that it is a valid option for MDR and XDR *P. aeruginosa* infections in cUTI, cIAI and VABP, with promising clinical success and limited treatment failure also described in SOT recipients. Further studies are needed to assess its efficacy, pharmacokinetics, and tolerability in this population.

## Ceftazidime/Avibactam (CZA)

### Activity Spectrum

CZA is a combination of the third-generation anti-pseudomonal cephalosporin ceftazidime and avibactam, a non-β-lactam BLI, which restores *in vitro* activity of ceftazidime against Ambler class A, class C and some class D (e.g., OXA-48) β-lactamases [33]; however, it remains inactive against metallo-β-lactamases (MBLs). To treat infections caused by bacteria with this latter resistance mechanism, CZA is used in combination with aztreonam to take advantage of its synergistic effect [34].

CZA is currently approved for treating cIAI, UTI and nosocomial pneumonia [35].

The licenced dose of CZA in patients with normal renal function is 2.5 g every 8 h, with dose reduction in patients with impaired renal function.

### Ceftazidime/Avibactam in Clinical Trial and its Potential Application in SOT Recipients

Data on using CZA in SOT recipients are limited to case reports and case series, mainly focusing on lung and kidney transplant recipients. Evidence in LTR is even scarcer and relies on retrospective real-world data analysis (Table 2). A Chinese case series of 21 LTR investigating the use of CZA in infections by KPC-producing Enterobacterales (KPC-E) [36] showed clinical response in adult patients at 14 days and 30 days of 70.6% (12/17) and 58.8% (10/17), respectively, while in paediatric patients was 75% at both time points. Three patients relapsed within 30 days. Most patients (66%) were treated with combination therapy (carbapenems, aztreonam, metronidazole, and polymyxin B), and no cases of CZA resistance were identified. Of note, three patients (3/21, 14.3%) developed acute kidney injury, and no other significant adverse event was reported. A similar study on six paediatric LTR [37] evaluated the efficacy and safety of CZA as salvage therapy for cIAI and bloodstream infections (BSI) caused by CRE, mostly KPC-E, and showed clinical success in all patients, without recurrence or development of resistance. CZA was mainly used as monotherapy (66%), and there were no serious adverse events.

**TABLE 2:** Overview of real-life studies describing ceftazidime/avibactam use among LTR.

Author, year	Country	Study design	Pathogen	Infection type	Main results	AE
Chen 2021 [36]	China	Retrospective observational study on 21 LTR (including 4 paediatric patients)	CRE KPC	IAI, BSI, PN	Mortality	3 (3/21, 14.3%) acute kidney injury, 2/21 patients received haemodialysis after CZA treatment
					• 14 days: 28.6%	Transient increase in ALT and AST blood levels was reported
					• 30 days: 38.1%	
					• All-cause: 42.9%	
					Clinical response	
					• Adult patients, 14 days: 70.6% (12/17); 30 days was 58.8% (10/17)	
					• Paediatric patients, both 14 days and 30 days: 75%	
					Relapse in 3 patients after 30 days	
					CZA resistance not detected in any case	
Wang 2022 [37]	China	Retrospective observational study on 6 paediatric LTR (≤12 years)	CRE KPC	IAI and BSI	Clinical success was achieved in all patients, no recurrences	Minor AE reported: vomiting (1/6), skin rash (1/6), increased GGT (2/6), (2/6), and alkaline phosphatase (3/6)
Di Pietrantonio 2022 [38]	Italy	Retrospective study on 81 pts receiving CZA for Gram-negative infections (8 LTR)	KPC	IAI, BSI, PN, VAP	Clinical failure for 7/8 (87.5%) patients	Not reported
					Significantly higher proportion of patients with clinical failure received LT (*p* = 0.003), mechanical ventilation (*p* = 0.049) or had pneumoniae (*p* = 0.009)	
					In multivariate logistic regression analysis, only LT is an independent predictor of treatment failure [OR 12.100 (1.369–106.971), *p* = 0.025]	
Perez-Nadales 2023 [39]	Spain, Italy, Brazil, United States	Retrospective study on 149 SOT recipients with KPC BSI (66 LTR)	KPC	BSI	Comparison between CZA and BAT.	Not reported
					Clinical success	
					• Day 14: CZA vs. BAT (80.7% vs. 60.6%)	
					• Day 30, CZA vs. BAT (97.4% vs. 60.6%)	
					All-cause mortality: CZA vs. BAT (13.3% vs. 27.3%)	

AE: adverse event; LTR: liver transplant recipient; CRE: Carbapenem-resistant Enterobacterales; IAI: intra-abdominal infection; BSI: bloodstream infection; PN: pneumonia; VAP: ventilator-associated pneumonia, CZA: ceftazidime-avibactam; BAT: best available therapy; LT: liver transplant; ALT: alanine transaminase; AST: aspartate aminotransferase; GGT: gamma-glutamyl transferase.

An international, retrospective cohort compared CZA with the best available therapy (BAT) in a cohort of 149 SOT recipients with KPC-Kp bloodstream infection (BSI) [39]. Liver (44.3%) and kidney (40%) were the most common SOT. Eighty-three patients received CZA, 37 of whom were LTR. Patients treated with CZA had a significantly higher rate of clinical success at day 14 than those treated with BAT (80.7% vs. 60.6%), particularly in the high mortality risk stratum according to the INCREMENT-SOT-CPE score [40]. The same trend was observed for clinical success at day 30, with significant differences observed between patients receiving CZA versus BAT in the treatment cohort. No stratification by SOT type was available.

Notably, CZA therapy was also associated with increased survival in the CAVICOR study, the most extensive series to date evaluating the impact of CZA on mortality in CRE infections. However, only 45/339 (13.2%) patients analysed were SOT recipients, and no stratification by SOT type was present [41].

In contrast, Di Pietrantonio et al. [38], analysing a cohort of 81 patients, 8 of whom were LTR, receiving CZA for infections mainly due to KPC-E, found that a significantly higher proportion of patients with clinical failure were LTR and that LTx emerged as an independent predictor of treatment failure. These differences may be due to the populations’ heterogeneity and the infection’s severity. Furthermore, the study was not designed to focus its analysis and results on a specific population such as LTR.

### Adverse Events and Limitations

Interactions with CZA and immunosuppressants are not expected, and no cases of induced hepatotoxicity have been reported in the Livertox database [42].

Monitoring renal function is warranted, especially when CZA is combined with other nephrotoxic molecules such as polymyxins or aminoglycosides.

### Key Messages

In conclusion, CZA may be a useful therapeutic option in LTR for treating infections caused by MDRGNB, particularly KPC-producing strains. New studies are needed to analyse the use of CZA in LTR, focusing on its efficacy versus BAT and examining its safety profile in this population. Caution is required in monitoring the emergence of CZA resistance during treatment of KPC-3-producing *K. pneumoniae*, as has already been reported [8, 9]. Finally, further evidence must be gathered on CZA combined with aztreonam for treating infections due to MBL-producing bacteria.

## Meropenem/Vaborbactam (MVB)

### Activity Spectrum

MVB is a new BL/BLI active on carbapenemases with a broad spectrum of enzyme inhibition. It combines meropenem (MEM), a carbapenem antibiotic, with vaborbactam, a highly specific BLI that targets KPC-β‐lactamase (including KPC-8 and KPC-3) and other class A beta-lactamases. In addition, combination with vaborbactam has been shown to reduce MEM minimum inhibitory concentration (MIC) in Enterobacterales with low MEM susceptibility harbouring ESBL or AmpC-type β‐lactamases [43, 44]. In contrast, MVB is inactive against class D or B carbapenemases [45]. The activity of MVB against other difficult-to-treat Gram-negative and anaerobic bacteria is variable: in general, the activity against *P. aeruginosa*, *Acinetobacter* spp., *Stenotrophomonas maltophilia* is comparable to that of MEM alone [46, 47].

### Meropenem/Vaborbactam in Clinical Trial and its Potential Application in SOT Recipients

Currently, two Phase 3 clinical trials have evaluated the efficacy and safety of MVB: the TANGO I [48] and TANGO II [49] studies. In the latter, immunocompromised patients, including SOT recipients, were enrolled, representing 32% of the total cohort and 40% of those with microbiologically confirmed CRE infection. Within the microbiologic carbapenem-resistant Enterobacterales modified intent to treat population, the cure rate was higher in the MVB group than in the BAT group at both the end of treatment and test of cure (65.6% vs. 33.3% and 59.4% vs. 32.7%, respectively). Despite not reaching statistical significance, mortality at 28 days was numerically lower with MVB than with BAT. The favourable outcome with MVB treatment is also confirmed when considering different infection categories. However, few patients in this cohort had cIAI (4, 8.5%), which limits the transferability of the results in the liver transplantation setting. Again, in additional subgroup analysis in immunocompromised patients, MVB had a higher cure rate at test of cure than BAT (63% vs. 0%). Overall, in this study, MVB emerged as an interesting treatment for CRE infection among LTR, although details on the type of SOT and immunosuppression were not specified.

A few case reports have demonstrated the use of MVB in clinical practice in LTR. One case report described MVB as salvage therapy for CZA-resistant *K. pneumoniae* abdominal abscess in an LTR [50]. The authors described an LTR with KPC-Kp BSI in the early post-transplant period, cured with CZA. Subsequently, the patient had a new BSI with an onset of *de novo* CZA resistance requiring discontinuation of CZA treatment, initiation of tigecycline and polymyxin B followed by gentamicin. Blood cultures were cleared, but CZA-resistant *K. pneumoniae* was recovered from the abscess fluid. MVB was initiated with complete recovery, allowing re-transplantation in the following days. In this case, MVB was efficacious in infection with a high bacterial inoculum.

Shield et al. [51], in 2019, described the use of MVB in 20 patients 11% SOT, type not specified and reported only in abstract presentation [52] with Enterobacterales infections, reporting KPC production in 90% of isolates. Survival rates at 30 and 90 days were 90% and 80%, respectively, and success rates were 63% in patients with BSI and 67% in patients with pneumonia. Clinical success was achieved in 65% (13/20) of patients. A significant rate of microbiologic failure was observed (6/20; 35%) due to recurrent CRE infection, respiratory colonisation, breakthrough during treatment, and persistent BSI. In two cases, microbiologic failure was associated with intra-abdominal abscess. In 50% of cases of recurrence, MIC for MVB increased significantly, and KPC-3 *K. pneumoniae* isolated in patients with intra-abdominal infection also acquired resistance to MVB. This point is relevant for LTR, where abdominal abscesses are frequent and may create an environment favourable for selecting antibiotic-resistant strains.

### Adverse Events and Limitations

Regarding adverse events (AE), in the TANGO I trial [48], patients in MVB discontinued treatment in 2.6% of cases because of AE. The most common AE reported was headache (8.8%), and liver toxicity was reported in a low percentage of cases (1.5%). In the TANGO II trial [49], AE associated with MVB included diarrhoea, anaemia, and hypokalaemia. Interestingly, MVB treatment experienced a lower level of renal insufficiency than BAT. A lower incidence of renal insufficiency was also described when MVB was compared to CZA [53]. No other side effects have been reported in studies of this drug. In addition, there are no known interactions with immunosuppressive medications, but real-life experience is needed to understand mechanisms better.

### Key Messages

MVB use in LTR is promising, especially for its anti-KPC activity, but more real-world data are needed. Its use in infections with high bacterial inoculum, requiring prolonged antibiotic therapies and source control, will require further investigation. In this setting, the toxicity of prolonged exposure and the potential development of resistance must be evaluated. In addition, more data are needed on interactions with immunosuppressive drugs.

## Imipenem/Relebactam (I-R)

### Activity Spectrum

-R is a new drug that is an intravenous combination of imipenem/cilastatin and relebactam, a non-β-lactam BLI. Relebactam (REL) is an inhibitor of class A and C β-lactamases [54]. Although REL has no intrinsic antibacterial activity, it can protect imipenem from degradation by Ambler class A and class C β-lactamases and *Pseudomonas*-derived cephalosporinase [55]. Instead, REL is inactive against class B MBLs or D oxacillinases [56, 57]. In addition, some *in vitro* studies have shown that REL is unaffected by efflux pumps at basal level of expression and does not suffer from inoculum effect [58].

### Imipenem/Relebactam in Clinical Trial and its Potential Application in SOT Recipients

There is a lack of data on using I-R in LTR [59]. I-R has been evaluated in two phase-2 clinical trials, two phase-3 clinical trials and a small amount of real-world clinical experience, but LTR and SOT were usually excluded.

Phase 2 clinical trials evaluated I-R in cases of cIAI [60] and cUTI [61] and demonstrated a favourable clinical response in both cases. However, the phase 3 studies raise interesting questions regarding the efficacy in SOT recipients. In RESTORE-IMI 1 [62], which compared the efficacy and safety of I-R versus colistin (COL) plus IMP in patients with IMP-susceptible hospital-acquired or ventilator-associated pneumonia (HAP/VAP), cUTI or cIAI, favourable overall responses were achieved in both arms (I-R, 71%; COL + IMP, 70%). Only patients with HAP/VAP and cUTI, but none with cIAI, achieved a favourable overall response. Of note, this data is biased by the small number of patients with cIAI enrolled [4], with one out of two patients in both arms experiencing an unfavourable overall response due to missing/undefinable data.

In addition, the recent RESTORE-IMI 2 study [63] evaluated I-R versus piperacillin-tazobactam (TZP) in patients with HAP/VAP. Unfortunately, immunocompromised patients were excluded per protocol, limiting the applicability of the study results to the LTR population. Overall, I-R was non-inferior to TZP for the primary (28-day all-cause mortality) and secondary endpoint (favourable clinical response at the end of follow-up). In a subgroup of patients with severe disease, 28-day mortality and end-of-treatment cure were higher in patients treated with I-R. In addition, patients with *P. aeruginosa* infection had a lower clinical response rate and higher 28-day mortality rate in the I-R arm, although both treatment arms had comparable microbiologic eradication rates at the end of treatment (67% I-R vs. 72% TZP) [62–65].

Few studies have published real-world experience with I-R. Konho et al. [64] described the experience with I-R in patients with cIAI and cUTI infections and evaluated safety and efficacy. They enrolled 83 patients (cIAI = 39, cUTI = 44). Adverse events occurred in 74.1% of cases, the most common being diarrhoea. Four patients discontinued treatment due to AE, but no serious AE was considered related to the study treatment. A favourable clinical and microbiological response was achieved in 85.7% of patients with cIAI at the end of treatment and 82.1% at the test of cure visit (5–9 days and 14 days after completion of treatment). Microbiologic response was achieved in all patients with cUTI at the end of treatment and 59% at the test of cure visit. Of 16 cUTI patients with an unfavourable microbiological response, 13 had a favourable clinical outcome.

The last real-world evidence study described the emergence of resistance to I-R in patients with *P. aeruginosa* HAP/VAP treated with this molecule [65]. The main observation was that 5 of 19 patients had the emergence of I-R non-susceptible *P. aeruginosa* during treatment or within 30 days after treatment. All five patients had failed prior antibiotic regimens, including two who received I-R after treatment-emergent resistance to C/T. At whole-genome sequencing, the *P. aeruginosa* isolate did not harbour MBLs or other ß-lactamase enzymes conferring resistance to I-R. However, in all patients, I-R non-susceptibility coincided with the emergence of mutations in *P. aeruginosa* efflux operons. In two patients, the *P. aeruginosa* strains were ST235 and ST244, known to be high-risk MDR clones [66]. All these mutations occurred during antibiotic treatment between 8 and 23 days of therapy, resulting in a shift of the I-R MIC to higher values. Further studies in real-life settings with patients with multiple comorbidities and a variety of potential drug interactions are needed to define the role of I-R in *P. aeruginosa* infections occurring among LTR.

### Adverse Events and Limitations

Regarding AE, similar data were reported in the available studies. In phase 2 [60] and phase 3 studies [62, 63], the most common AE were nausea, diarrhoea, and elevated liver enzymes. Focusing on liver toxicity, in RESTORE IMI-1, the incidence was between 2% and 3%, while in RESTORE IMI-2, the incidence was 2.3% [62, 63]. In general, in the RESTORE IMI-1 study, three patients (19%) in the COL + IMP arm and none in the I-R arm discontinued treatment due to AE, while in the RESTORE IMI-2 study, six patients (2.3%) in the I-R arm and four (1.5%) in the TZP arm discontinued treatment due to drug toxicities [62, 63].

Regarding renal toxicity, I-R was associated with a more favourable renal safety profile than COL-based therapy in RESTORE IMI-1. These data were also confirmed by a subsequent retrospective study conducted with RESTORE IMI-1 data using two assessment criteria for acute kidney injury, strengthening, as expected, how I-R had a better safety profile than IMP-COL [62].

Concerning drug interactions, it is essential to know that I-R may interact with other antimicrobial and antiviral treatments. The use of I-R with amikacin, azithromycin, aztreonam, COL, gentamicin, levofloxacin, linezolid, tigecycline, tobramycin, or vancomycin has been tested, and it is allowed. Instead, I-R should not be used concomitantly with ganciclovir due to the increased risk of seizures unless the potential benefit outweighs the risk [67]. Given the many concomitant medications LTR need, more data on this issue is needed.

### Key Messages

I-R could be a promising drug in the LTx setting, mainly because of its broad spectrum of activity, covering anaerobes, *Enterococcus faecalis*, Enterobacterales and *P. aeruginosa* strains, even in the MDR setting. This feature is handy in intra-abdominal infections, a frequent complication after LTx. However, several issues remain to be clarified—first, the efficacy and emergency of non-susceptible I-R strains. LTR have often experienced multiple lines of antibiotic treatment, are often colonised or infected by MDRGNB, and sometimes experience deep infections requiring source control and prolonged antibiotic therapy. Knowing whether exposure to antibiotics could select for resistant strains is critical in this setting. Second, the liver toxicity described in RESTORE-IMI 2 needs to be investigated in-depth, and drug-drug interactions, especially with immunosuppressive treatment, need to be evaluated, given the higher rate of interactions with other molecules. Specifically, the contraindication to use ganciclovir concomitantly may be a limitation in this setting, given the frequent, ongoing treatment for CMV.

## Cefiderocol (FDC)

### Activity Spectrum

FDC is a novel siderophore cephalosporin antibiotic that is indicated for treating infections due to aerobic Gram-negative organisms in adults with limited treatment options [68]. FDC bind to free iron molecules, and it is actively transported across the outer membrane of bacteria by their iron-transport system, thus leading to the accumulation of the antibiotic inside the microorganism [69]. Exploiting this strategy, FDC can overcome resistance mechanisms due to efflux pumps, particularly common in MDRGNB such as *P. aeruginosa* [70]. Moreover, FDC potent activity against MDRGNB is also related to its high stability against various extended-spectrum-lactamases (ESBLs) and carbapenemases [71]. Clinical data for FDC are promising, with several studies demonstrating its efficacy in treating various infections caused by multidrug-resistant bacteria, including cUTI, HAP, and BSI [72–74]. Notably, FDC displayed a significant activity in infections due to MBL-producing bacteria, a condition with minimal therapeutic opportunities [75].

### Cefiderocol in Clinical Trial and its Potential Application in SOT Recipients

Regarding LTx, there is limited data on using FDC, with all data coming from case reports/series.

In their case series of difficult-to-treat infections due to MDRGNB treated with FDC, Bavaro et al. [76] included one LTR who received a combination therapy with FDC plus COL plus tigecycline followed by FDC plus fosfomycin for CZA-resistant KPC-Kp strain, causing liver abscess with bloodstream involvement. FDC was administered for 28 days, with a successful clinical outcome.

Klein et al. [77] reported the case of an LTR who underwent re-transplantation 10 years after receiving the first graft and who had a complicated clinical course with carbapenem-resistant *Enterobacter cloacae* BSI, initially treated with MEM and COL and subsequently with FDC alone. Within 21 days of therapy, the germ became resistant to FDC, and the patient died due to uncontrolled infectious focus.

Bodro et al. [78] presented instead a case of persistent BSI related to an infected transjugular intrahepatic portosystemic shunt caused by an extensively drug-resistant *P. aeruginosa*, resistant to ceftazidime, C/T, and MEM in a kidney transplant recipient who subsequently underwent a combined kidney-liver transplant. The patient received initial combination therapy with FDC plus COL for 2 weeks, followed by FDC alone for 4 weeks, resolving the infection.

### Adverse Events and Limitations

Limited information regarding potential drug interactions between FDC and immunosuppressive drugs commonly used in liver transplantation is available. FDC is primarily eliminated unchanged in the urine and is not extensively metabolised by the liver [68]. As such, the risk of significant drug interactions with immunosuppressive drugs primarily metabolised by the liver may be low. In the CREDIBLE-CR study, liver-related adverse events (specifically increased liver enzyme concentrations) were reported more frequently in patients treated with FDC than with the best available therapy. It should be noted how the study included a relevant number of patients with ongoing hepatic disease (moderate/severe liver disease 11/101, hepatitis 12/101), how the adverse events were of mild/moderate severity and transient in duration and how no cases met the clinical and biochemical criteria for Hy’s law or drug-induced liver injury [74]. Instead, in the APEKS-NP study, no notable differences between the treatment groups (MEM vs. FDC) were identified in the occurrence of liver-related adverse events. In contrast, in the NCT02321800 trial (a multicenter, double-blind, randomized clinical study to assess the efficacy and safety of FDC in hospitalized adults with cUTI caused by Gram-negative pathogens), liver-related adverse events were not described [72, 73]. Of note, currently, there are no reported cases of liver toxicity due to FDC reported in the Livertox database [79].

FDC may cause renal impairment, which could be exacerbated by the concomitant use of nephrotoxic drugs commonly used in liver transplantation, such as calcineurin inhibitors (e.g., tacrolimus, cyclosporine) [80]. Therefore, it may be necessary to monitor renal function closely and adjust the dose of immunosuppressive drugs accordingly [68].

Finally, therapeutic and supratherapeutic doses of FDC had no apparent clinically significant effect on the QTc. Thus, no specific monitoring with electrocardiography is required during FDC therapy [81].

### Key Messages

Overall, while there is limited data specifically on the use of FDC in liver transplantation, the available evidence suggests that it may be a safe and effective treatment option for multidrug-resistant infections, especially when due to MDRGNB harbouring MBLs and *P. aeruginosa* DTR. However, further studies are needed to confirm these findings and evaluate its optimal employment in this patient population.

## Eravacycline (ERV)

### Activity Spectrum

ERV is a novel, fully synthetic fluorocycline belonging to the tetracycline class. It has a broad-spectrum activity against aerobic and anaerobic Gram-positive and Gram-negative microorganisms and was explicitly designed to maintain stability against efflux pumps and ribosomal protection proteins. ERV is active against various MDR pathogens such as methicillin-resistant *Staphylococcus aureus* strains, vancomycin-resistant *Enterococcus faecium*, *Enterococcus faecalis*, ESBL-E, and AmpC-producing Enterobacterales [82]. On the other hand, it shows limited activity against *P. aeruginosa* and *Stenotrophomonas maltophilia*.

ERV exerts its antimicrobial action by primarily binding to the ribosomal 30 s subunit, interrupting the elongation phase of protein synthesis. *In vitro* shows a ten-fold higher activity at a four-fold lower drug concentration than other tetracyclines. Similarly, data from the CANWARD surveillance study demonstrated that ERV carries an *in vitro* activity equivalent to or 2- to 4-fold greater than tigecycline against Enterobacterales and Gram-positive bacteria [83].

### Eravacycline in Clinical Trial and its Potential Application in SOT Recipients

ERV has been approved in several countries, including the EU and United States, for treating adult patients with cIAI. Two randomised, double-blind, non-inferiority phase 3 trials [84, 85] evaluated its efficacy in treating subjects with cIAI, acknowledging this drug as non-inferior to intravenous ertapenem or MEM, respectively, at the test-of-cure visit in terms of clinical response rates in all prespecified populations. Unfortunately, none of the trials included data on the efficacy of ERV in treating CRE and/or MDR *Acinetobacter spp*.

ERV has also been investigated in cUTI: two trials compared it with ertapenem and levofloxacin, respectively, reporting lower cure rates [86, 87]. In this setting, its use is not recommended.

Regarding treating infections in the setting of LTx, there is still no specific data on the use of ERV. A recent retrospective, multicentre study evaluated ERV clinical use in a cohort of 66 patients with infections by MDRGNB or Gram-positive cocci, with 7 of them being SOT recipients. Most subjects received the drug for an off-label indication, and overall, a good clinical response was reported (63/66 patients, 95.5%) [88].

### Adverse Events and Limitations

There is limited information regarding potential drug interactions between ERV and immunosuppressive drugs commonly used in LTx. The absence of data is supported by the fact that clinical trials did not include immunosuppressed subjects. ERV is metabolised by liver cytochrome CYP3A4 and flavin-containing monooxygenase and excreted in urine and faeces. Therefore, concomitant administration of immunosuppressive drugs generally metabolised by the liver should be considered and closely monitored. Both the Food and Drug Administration and the European Medicines Agency suggest increasing ERV dose when co-administered with strong CYP3A4-inducers; on the other hand, coadministration with CYP3A4-inhibitors (e.g., tacrolimus) is not likely to cause a clinically significant increased exposure. Moreover, *in vitro*, ERV has been displayed to be a substrate for the transporters P-gp, OATP1B1 and OATP1B3. This kind of interaction cannot be excluded *in vivo*, and therefore, coadministration of ERV with drugs that inhibit these transporters (e.g., cyclosporine) could increase ERV serum levels [89].

Regarding side effects, ERV has demonstrated an acceptable tolerability profile, with infusion site reactions, nausea, vomiting, and diarrhoea being the most common AE.

Regarding hepatotoxicity, data from preclinical trials report mild to moderate aminotransferase elevations. The literature does not report cases of drug-induced liver injury associated with ERV use [42].

Considering the described elimination and excretion features, ERV does not seem to cause renal impairment.

Therapeutic and supra-therapeutic doses of ERV had no apparent clinically significant effect on electrocardiographic traces (e.g., increase in QTc interval); thus, no specific monitoring with electrocardiography is required during ERV therapy [90].

### Key Messages

Overall, while there is still no data on the specific use of ERV in LTx, the available evidence in the setting of cIAI and the peculiar drug features suggest that it may be a safe and effective treatment option for infections caused by difficult-to-treat bacteria. However, studies are needed to confirm these findings and evaluate its optimal employment in this patient population.

### PK/PD of New Molecules

All the above-described antibiotics, except ERV, belong to the beta-lactam class. They all demonstrate time-dependent killing with the PK/PD parameter of efficacy related to the amount of time that the unbound drug concentration remains above the MIC of the infecting organism (*f*T_>MIC_) [91]. It is suggested that 50% *f*T_>MIC_ is likely enough to obtain standard efficacy, while in critically ill immunocompromised individuals, up to 100% *f*T_>4 x MIC_ should be ensured for optimal drug exposure and suppression of resistance development [92–94].

LTx candidates with end-stage organ failure and SOT patients in the early post-operative period are characterised by profound pathophysiological alterations that resemble those of critically ill patients. These alterations significantly impact the PK/PD of BLs [91, 92, 94, 95]. Indeed, conditions frequently encountered in the LTx period could either increase Vd (capillary leakage and oedema, fluid therapy, ascites, hypoalbuminemia) or enhance renal clearance (hyperdynamic condition of the early phase of sepsis, use of hemodynamically active drugs), leading to the risk of drug underdosing. On the contrary, reduced renal clearance due to renal failure bedridden or concomitant nephrotoxic drugs may expose them to antibiotic overdosing and toxicity [91, 92, 94, 95]. Extracorporeal support techniques such as continuous renal replacement therapy (CRRT) also contribute to antibiotic concentration variability [95]. In addition, critically ill patients with decompensated cirrhosis have a unique PK variability that can affect serum drug concentrations and compromise target attainment. Severe acute on chronic liver failure (ACLF) frequently presents circulatory and renal dysfunctions and low serum protein levels, features that contribute to ascites and frequently anasarca. This setting will likely significantly affect both clearance and Vd of antibiotics. Population PK models have shown that increasing MELD score values reduce MEM and tigecycline clearance, demanding a reduction in drug doses [96, 97]. ACLF was found to increase MEM Vd by lowering peak concentrations. Consequently, higher loading doses of MEM have been suggested [97].

Clinicians must face these remarkable PK/PD issues when antibiotics are administered to critically ill patients. Strategies to overcome these issues and optimise beta-lactam efficacy include continuous/extended infusions (C/EI) and therapeutic drug monitoring (TDM).

The duration of BL infusion has been shown to influence their *f*T _>MIC_ [94]. Several studies and systematic reviews reported PD benefits for target attainment of C/EI of beta-lactams, especially in infections by MDRGNB [98–101].

Vardakas et al. [98] conducted a meta-analysis of 22 randomised controlled trials comparing C/EI versus short-term infusion of antipseudomonal beta-lactams in sepsis, involving 1876 patients. C/EI was associated with lower all-cause mortality than short-term infusion (RR 0.70, 95% CI 0.56–0.87). Almost all subgroup and sensitivity analyses showed that C/EI was associated with at least a trend towards lower all-cause mortality than short-term infusion [98].

Bartoletti et al. [101] performed a secondary analysis of the BICHROME study, a prospective multicentre study conducted in 19 tertiary centres across five countries designed to describe the epidemiology of BSI in cirrhotic patients. The authors reviewed 119 patients treated with TZP or carbapenems as empirical treatment and observed a significantly lower mortality rate in those who received C/EI (after adjusting for severity of illness: HR 0.41, 95% CI 0.11–0.936). A significant reduction in 30-day mortality was also found in the subgroups of patients with sepsis (HR 0.21, 95% CI 0.06–0.74), acute-on-chronic liver failure (HR 0.29, 95% CI 0.03–0.99), and MELD score ≥25 (HR 0.26, 95% CI 0.08–0.92) [101].

Among novel beta-lactams, EI was considered in developing clinical trials only for MVB and FDC, which nowadays are the only two novel beta-lactams licensed to be administered by EI over 3 h. However, administration by intermittent infusion could lead to failure in achieving even the most conservative PK/PD target adopted in pivotal trials, especially in critically ill patients or infections by MDRGNB [100]. Real-world evidence on using novel beta-lactam antibiotics by C/EI in clinical scenarios when achieving aggressive PK/PD targets is challenging has been thoroughly reviewed [99, 100].

TDM had been historically instituted for aminoglycosides and glycopeptides to reduce the rate of drug toxicity. Because of the excellent safety profile of BLs, TDM was thought unnecessary for these antibiotics. More recently, challenges in achieving “optimal” drug concentrations in critically ill patients have suggested BL TDM as a valuable strategy to optimise PK/PD exposure, especially in infections by MDRGNB, immunocompromised patients and those undergoing CRRT or with augmented renal clearance [92, 95].

Focusing on critically ill patients with suspected or proven sepsis, Pai Mangalore et al. [102] conducted a systematic review and meta-analysis on TDM-guided dosing and clinical outcomes. TDM-group was associated with increased target attainment (RR 1.85, 95% CI 1.08–3.16) and improved clinical cure (RR 1.17, 95% CI 1.04–1.31), microbiological cure (RR 1.14, 95% CI 1.03–1.27) and treatment failure (RR 0.79, 95% CI 0.66–0.94) [102].

Table 3 summarises scheduled and suggested administration modalities for maximising novel BL antibiotics’ PK/PD target, focusing on the LTx setting.

**TABLE 3:** Suggested dosages and infusion modalities for maximising PK/PD target of novel antibiotics, with particular focus to the LTx setting. Adapted from [99, 100].

Antibiotic	PK/PD target adopted in pivotal trials	Scheduled infusion modality	Optimised PK/PD target (maximise efficacy, suppress resistance development)	Stability in solution	Suggested dosage for maximising PK/PD target^a^	Considerations for LTx setting
Ceftolozane/tazobactam [103]	30% *f*T_>MIC_	II over 1 h	100% *f*T_>4 x MIC_	24 h	LD: 2 g/1 g	• negligible hepatic metabolism, not expected to be affected by hepatic impairment. No dose adjustment recommended as per SPC
					MD: 2 g/1 g q8h CI	• TDM-guided approach may be useful in ACLF and/or high MELD score
Ceftazidime/avibactam [104]	50% *f*T_>MIC_	II over 2 h	100% *f*T_>4 x MIC_	12 h	LD: 2 g/0.5 g	• no relevant hepatic metabolism. No dose adjustment as per SPC (no PK data of ceftazidime in patients with severe hepatic impairment; no PK data of avibactam in patients with any degree of hepatic impairment)
					MD: 2 g/0.5 g q8h CI	• TDM-guided dose should be obtained in deep-seated infections, ACLF and/or high MELD score
Meropenem/vaborbactam [105]	45% *f*T_>MIC_	EI over 3 h	100% *f*T_>4 x MIC_	12 h	LD: 2 g/2 g	• no relevant hepatic metabolism. No dose adjustment as per SPC (hepatic function monitoring recommended in patients with pre-existing liver disorders due to the risk of hepatic toxicity)
					MD: 2 g/2 g q8h CI	• TDM-guided dose should be obtained in ACLF and/or high MELD score
Imipenem/relebactam [106]	40% *f*T_>MIC_	II over 0.5 h	100% *f*T_>4 x MIC_	3.5 h	500 mg/250 mg q6h EI over 3 h	• no relevant hepatic metabolism. No dose adjustment as per SPC (hepatic function monitoring recommended in patients with pre-existing liver disorders due to the risk of hepatic toxicity)
						• TDM-guided dose should be obtained in ACLF and/or high MELD score
Cefiderocol [89]	75% *f*T_>MIC_	EI over 3 h	100% *f*T_>4 x MIC_	6 h	LD: 2 g	• no relevant hepatic metabolism. No dose adjustment as per SPC
					MD: 2 g q8h CI	• TDM-guided approach may be useful in ACLF and/or high MELD score
Eravacycline [89]	*f*AUC/MIC ratio	II over 1 h	N/A	12 h	as per SPC	• No dose adjustment as per SPC
						• Exposure may be increased in patients with Child-Pugh Class C (twofold increase in AUC, half-life prolonged from 16 to 21–26 h), particularly if obese and/or also being treated with potent CYP3A inhibitors. In these patients, no recommendation on posology given
						• TDM-guided approach not available

LTx: liver transplant; PK/PD: pharmacokinetic/pharmacodynamic; MIC: minimum inhibitory concentration; II: intermittent infusion; EI: extended infusion; CI: continuous infusion; LD: loading dose; MD: maintenance dose; SPC: summary of product characteristics (EMA); TDM: therapeutic drug monitoring; ACLF: acute on chronic liver failure; MELD: Model for End-Stage Liver Disease.

^a^ For patients with normal renal function.

## Conclusion

Overall, the scientific and clinical community has warmly received the availability of a discrete number of new molecules active against MDRGNB, as it represents a significant breakthrough in addressing the urgent need for effective antibiotics in the face of rising antimicrobial resistance. This holds particularly true within the setting of LTx, wherein the prevalence of infections caused by MDRGNB is considerable, and patients undergo extensive surgical procedures while concurrently receiving immunosuppressive therapy.

Despite the high anticipation surrounding the introduction of these medications, substantial evidence regarding their safety, effectiveness, and optimal utilisation in LTR is limited or lacking. Given the underrepresentation of this patient population in conventional registration studies, the transplantation community must collaborate to collect the necessary data to optimise their usage.

## Figures and Tables

**Table:** 

**ACLF**	acute on chronic liver failure
**AE**	adverse events
**BAT**	best available therapy
**BL**	beta-lactam
**BLI**	beta-lactamase inhibitor
**BSI**	bloodstream infection
**C/EI**	continuous/extended infusions
**C/T**	ceftolozane/tazobactam
**cIAI**	complicated intra-abdominal infections
**COL**	colistin
**CRAB**	carbapenem-resistant *Acinetobacter baumannii*
**CRRT**	continuous renal replacement therapy
**CRE**	carbapenem-resistant Enterobacterales
**cUTI**	complicated urinary tract infections
**CZA**	ceftazidime/avibactam
**ESBL-E**	ESBL-producing Enterobacterales
**ESBL**	extended-spectrum-β-lactamases
**ERV**	eravacycline
**FDC**	cefiderocol
**HAP**	hospital-acquired pneumonia
**I-R**	imipenem/cilastatin/relebactam
**IMP**	imipenem
**LTR**	liver transplant recipients
**LTx**	liver transplantation
**KPC-E**	KPC-producing Enterobacterales
**MBL**	metallo-β-lactamases
**MDR**	multidrug-resistant
**MDRGNB**	multidrug-resistant Gram-negative bacteria
**MEM**	meropenem
**MIC**	minimum inhibitory concentration
**MVB**	meropenem/vaborbactam
**PK/PD**	pharmacokinetic/pharmacodynamic
**REL**	relebactam
**SOT**	solid organ transplant
**TDM**	therapeutic drug monitoring
**TZP**	piperacillin-tazobactam
**VABP**	ventilator-associated bacterial pneumonia
**VAP**	ventilator-associated pneumonia
